# Exploring the link: Sturge-Weber syndrome and pituitary macroadenomas: A case report and review

**DOI:** 10.1016/j.radcr.2024.07.115

**Published:** 2024-08-18

**Authors:** Razi Hashmi, Mohamed Elmeligy, Daniel Fabian, Arun Mahtani, Meena Farid, Meheret Kinfe, Paul Karroum, GianPaolo Piccione, Mahmoud Mahmoud, Mohamed Albakri, Inderbir Padda

**Affiliations:** a Department of Internal Medicine, Richmond University Medical Center/Mount Sinai, Staten Island, NY, USA; b Department of Radiology, Richmond University Medical Center/Mount Sinai, Staten Island, NY, USA; c Department of Medicine, American University of Antigua, Staten Island, NY, USA

**Keywords:** Endocrinology, Neurology, Pituitary Macroadenomas, Seizure, Sturge-Weber Syndrome

## Abstract

Sturge-Weber syndrome (SWS) is a rare congenital disorder marked by facial port-wine birthmarks, neurological impairments, and ocular anomalies. Our case report describes a 20-year-old man with SWS who presented with right-sided weakness, slurred speech, and oral dyskinesia. Laboratory studies demonstrated elevated prolactin levels, and imaging confirmed a pituitary macroadenoma. While not well-documented, the association between SWS and pituitary macroadenomas is emerging based on current evidence. The potential link may involve embryological, genetic, or hormonal factors influencing the simultaneous development of these conditions. This case highlights the need for a thorough evaluation in patients with SWS, incorporating both neuroimaging and endocrine assessments to manage associated complications effectively. Further research is necessary to investigate the link between SWS and pituitary tumors. Establishing evidence-based guidelines for the screening and management of these patients will improve outcomes and provide a standardized approach to care.

## Introduction

Sturge-Weber syndrome (SWS), also known as encephalotrigeminal angiomatosis, is a rare congenital disorder that primarily affects the skin, brain, and eyes. It is characterized by the presence of port-wine birthmarks (capillary malformations), neurological, and ocular abnormalities [1]. SWS does not follow any inheritance pattern and occurs sporadically due to a somatic mutation in the GNAQ gene, affecting development of blood vessels [2]. SWS can also present with a wide variety of neurological manifestations, with epilepsy being the most common affecting 75%-90% of the cases [3]. Seizures usually begin in infancy or early childhood and can pose significant management challenges, often necessitating the use of anti-epileptic medications. Vascular malformations in the brain can lead to impaired blood flow and elevated intracranial pressure. This can result in intellectual and developmental disabilities, as well as focal deficits leading to weakness or paralysis on one side of the body . Additional complications include glaucoma, migraines, and learning disabilities [4,5].

Given the prevalence of neurological symptoms associated with SWS, additional differential diagnoses must include neuroendocrine tumors, as highlighted in this case report. For example, prolactinomas, which account for up to 40% of all pituitary adenomas, represent the most prevalent cause of hyperprolactinemia [6]. Other potential causes of hyperprolactinemia include medications, pregnancy, renal failure, and hypothyroidism. The clinical features of hyperprolactinemia include hypogonadism, decreased libido, infertility, galactorrhea, gynecomastia, headaches, and visual field defects in males. Diagnosis involves measuring serum prolactin levels alongside a comprehensive hormonal profile. Imaging studies, particularly magnetic resonance imaging (MRI), are preferred for identifying the size and type of adenomas, with lesions larger than 1cm classified as macroadenomas [7,8]. The regulation of prolactin secretion is primarily mediated by dopamine from the hypothalamus, which inhibits prolactin release from the anterior pituitary. Additionally, prolactin levels have been noted to spike following seizure activity, peaking within 15-30 minutes postseizure, and this transient increase may serve as a biomarker for distinguishing between epileptic and psychogenic seizures [9]. Moreover, these fluctuations in prolactin could suggest seizure activity induced by SWS or hormonal changes due to a pituitary adenoma making it challenging to differentiate between the two.

## Case presentation

**Fig. 1:**
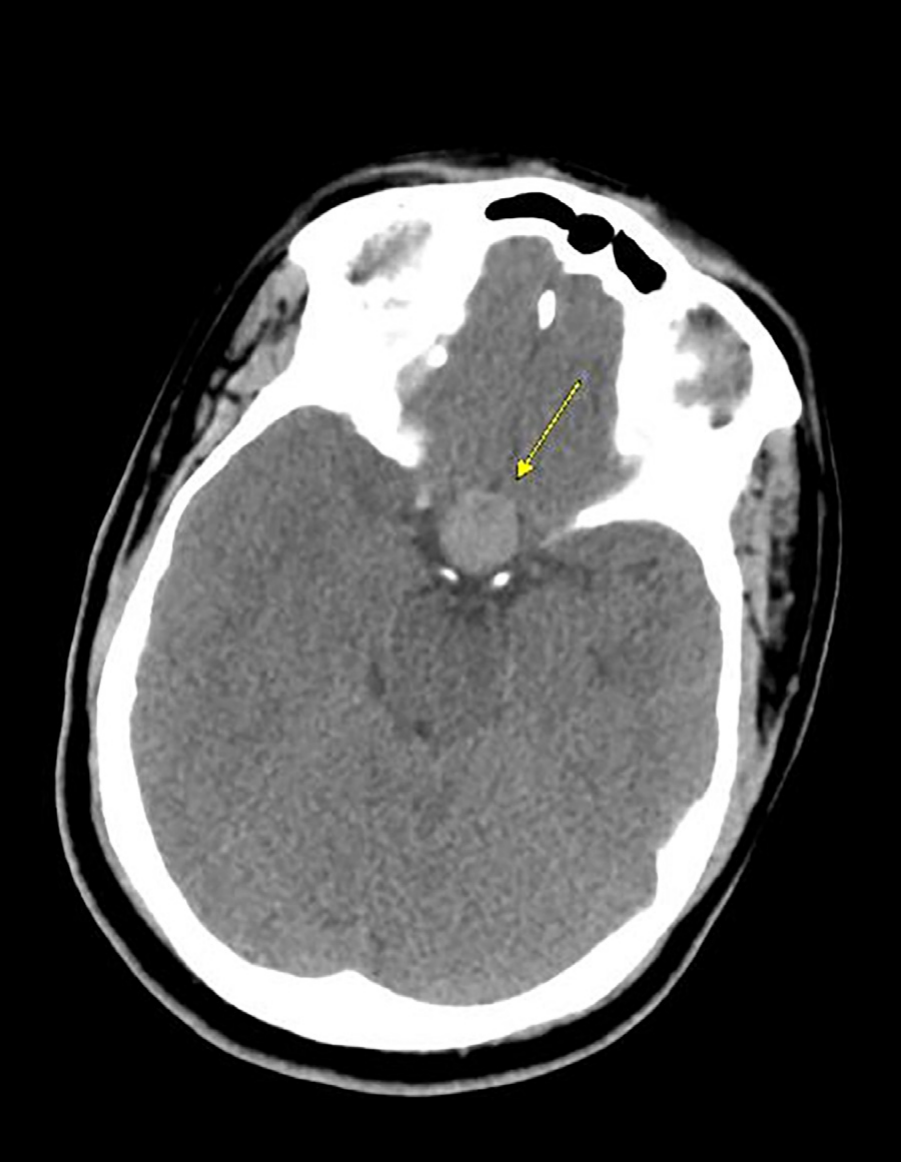
Initial imaging with Axial Noncontrast CT head taken June 2021 showing a heterogeneously hyperdense lesion **(yellow arrow)** in the suprasellar cistern with central hypodensity that appears to be extending from the sella turcica and measures approximately 1.8 × 1.7 × 3.0 cm, causing mass effect upon the optic chiasm. Differential at this stage includes pituitary macroadenoma, or less likely meningioma, or less likely aneurysm.

**Fig. 2:**
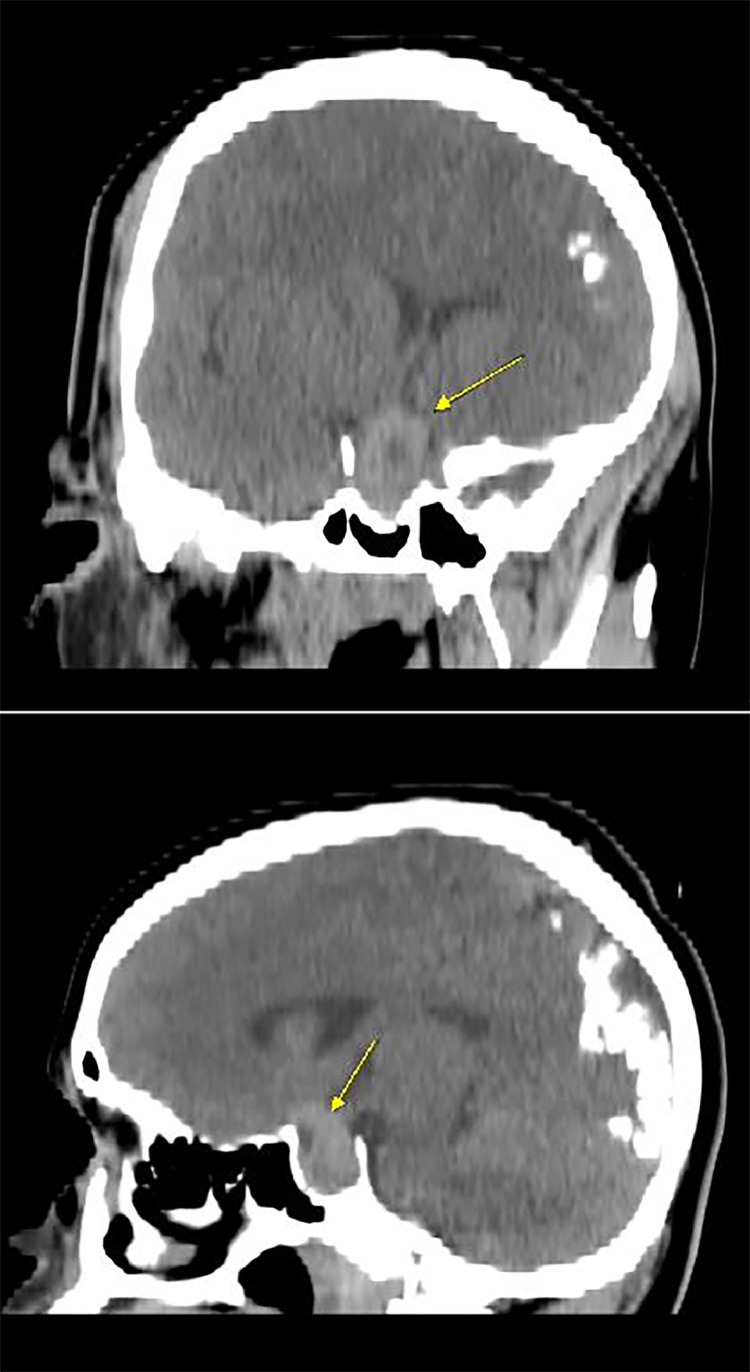
Initial sagittal and coronal images of previously described suprasellar lesion **(yellow arrow)** showing mass effect upon the optic chiasm and measuring up to 3cm in the craniocaudal direction.

**Fig. 3:**
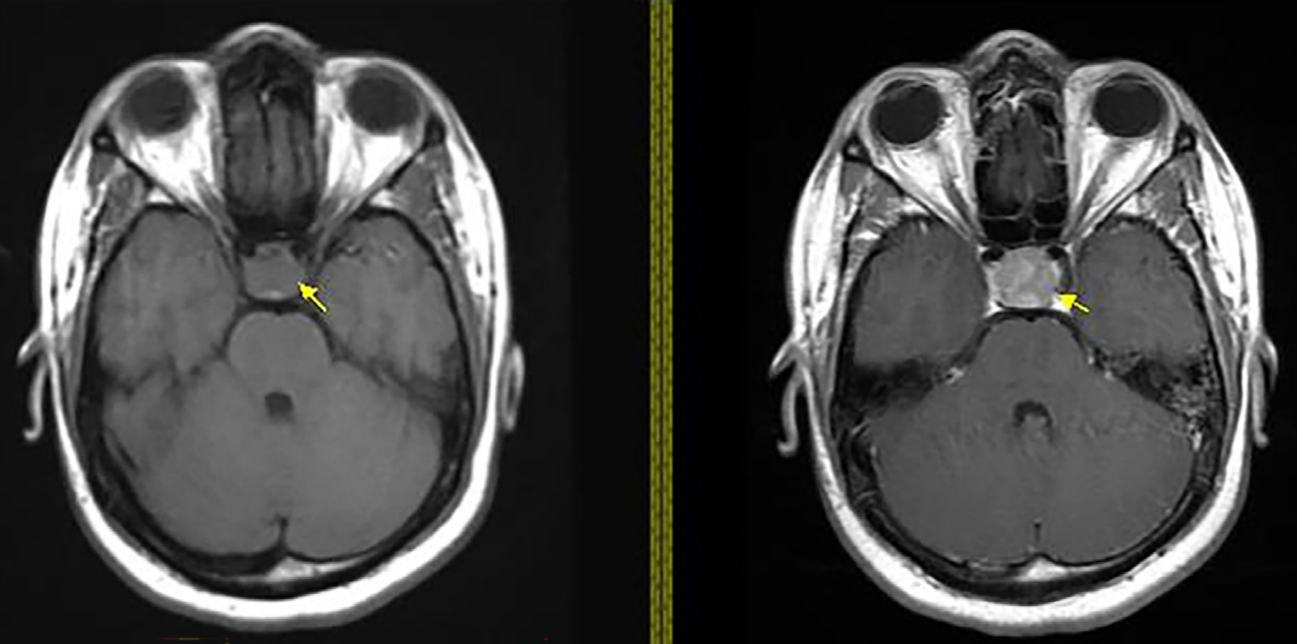
Initial Axial T1 MRI with **(right)** and without **(left)** contrast June 2021. Noncontrast image shows isointense lesion in the suprasellar region with small area posteriorly showing increased T1 signal. On contrast enhanced images this lesion shows heterogeneous enhancement most consistent with pituitary macroadenoma **(yellow arrow)**.

**Fig. 4:**
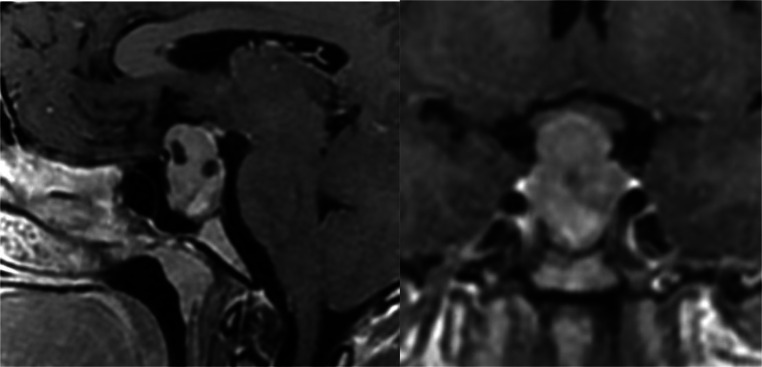
Zoomed in Sagittal and coronal postcontrast enhanced T1 imagers of said 3 cm sellar and suprasellar mass showing heterogenous enhancement with hypointense areas likely representing areas of cystic change with mass effect upon the optic chiasm causing superior displacement.

**Fig. 5:**
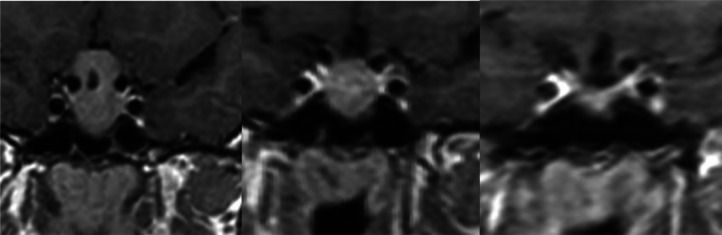
Zoomed in coronal postcontrast imaging showing progression of treatment of macroadenoma with initial **(left)** image showing a 3 cm mass in the sellar and suprasellar regions causing superior displacement of the optic chiasm, with subsequent **(middle)** imaging taken 3 months later showing significant decrease in size now measuring 1.5 cm in maximal diameter without suprasellar extension or compression of the optic chiasm. Final images taken 2 years later **(right)** shows complete resolution of mass without residual mass burden.

**Fig. 6:**
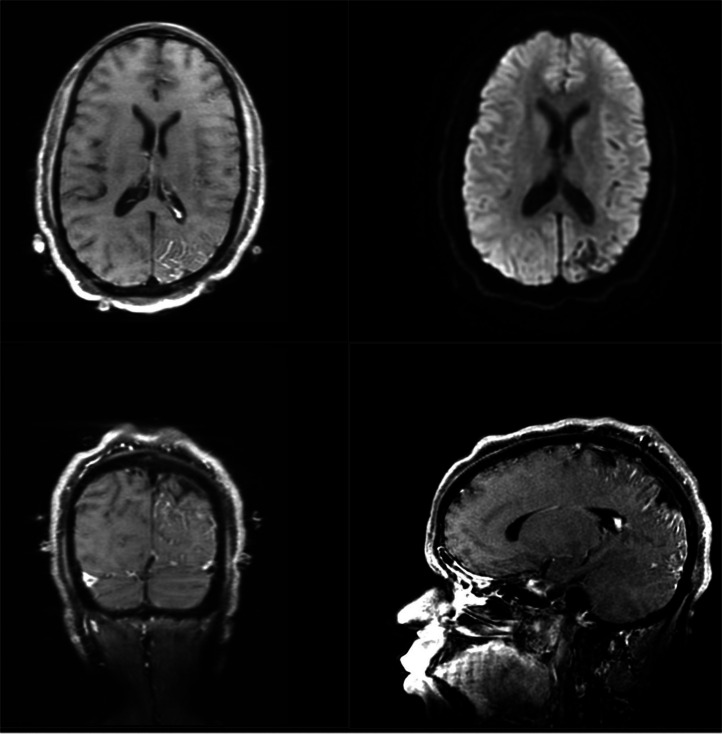
Axial DWI sequence **(top right)** and Axial Coronal and Sagittal T1 weighted Post contrast reformatted images showing tram-track gyriform calcification in the left parietal lobe with associated atrophy and intense gadolinium enhancement in the leptomeninges consistent with Sturge-Weber syndrome.

A 20-year-old male with a past medical history of SWS presented to the emergency department with a 2-day history of right-sided weakness, slurred speech, altered consciousness, and an episode of oral dyskinesia while playing basketball. Upon evaluation, the patient appeared somnolent but denied any head trauma related to the basketball game. He reported 2 recent episodes of vomiting and feeling cold but denied fevers, chills, headaches, blurry vision, hearing loss, facial droop, chest pain, or other related symptoms. His medical history also noted a history of seizures during childhood with the last episode occurring at age 12. Since then he had not taken any antiepileptic medications. Upon physical examination, the patient was alert and oriented to time, place, and person but exhibited slowed responses and slurred speech without facial droop. Extraocular movements were intact. The neurological assessment showed motor strength was graded as 3/5 in the right upper extremity, 5/5 in the left upper extremity, 4/5 in the right lower extremity, and 5/5 in the left lower extremity. Decreased tactile sensation was observed in both lower extremities up to the knees, and herpetic vesicles were noted on the lower lip. Preserved finger-to-nose coordination on the left side was noted with difficulty in the right arm. Laboratory findings indicated elevated prolactin levels > 200 Ng/mL. The CBC and CMP were within normal limits. Urinalysis was insignificant and urine drug screen (UDS) was positive for marijuana. Blood cultures and urine culture showed no bacterial growth. An electroencephalogram demonstrated moderate generalized background slowing, suggestive of diffuse cerebral dysfunction with nonspecific etiology, and possible sharp wave activity in the occipital region. Imaging with axial noncontrast computed tomography (CT) head showed a heterogeneously hyperdense lesion (yellow arrow) in the supra-cellar cistern with central hypodensity that appears to be extending from the sella turcica and measures approximately 3.0 cm, causing mass effect upon the optic chiasm consistent with a macroadenoma (Fig. 1, Fig. 2). The CT findings were then confirmed with an MRI with and without contrast (Fig. 3, Fig. 4, Fig. 5). The patient was initiated on antiepileptic treatment with levetiracetam. The neurosurgery team evaluated the patient, and no surgical intervention was planned for his macroadenoma. The patient was also found to have an elevated prolactin level > 200 ng/mL after 5 days of being seizure-free, prompting the initiation of cabergoline 0.5 mg twice weekly upon discharge, along with continued regimen of levetiracetam 1g twice daily for seizure prophylaxis. Follow-up CT scan of the head and MRI of the head after discharge revealed a reduction in the size of the mass by 1-cm at the 3 month interval and complete resolution of the mass at the 2-year follow-up (Fig. 5, Fig. 6).

## Discussion

As evidenced by our case report, there may lie a possible association between SWS and the development of pituitary macroadenomas. While this association is not well-documented, it raises important clinical considerations when treating patients with this underlying syndrome. The exact mechanisms underlying the association between SWS and pituitary macroadenomas are not fully understood. However, several theories may be proposed which include embryologic, genetic, and hormonal etiologies. SWS and pituitary macroadenomas may share common embryological origins. Disruptions in the development of blood vessels and tissues during fetal growth may lead to the concurrence of these conditions. There may also be genetic factors at play that predispose individuals with SWS to the development of pituitary tumors. Specific genetic mutations or alterations could increase the risk of developing both conditions. Imbalances or alterations in the hypothalamic-pituitary axis may also contribute to the development of macroadenomas in individuals with SWS, suggesting a hormonal component to this correlation. The potential association between SWS and pituitary macroadenomas has significant clinical implications. It highlights the importance of comprehensive medical evaluations for individuals with SWS, even when their primary symptoms are related to neurological or cutaneous manifestations. Early detection and management of pituitary macroadenomas are crucial to prevent potential complications such as hormonal imbalances, vision changes, neurological deficits or pituitary apoplexy leading to cerebral infarction rarely. [10] Approximately 18% to 78% of patients with macroadenomas endure visual field defects and around 34% to 89% are left with hypopituitarism in their life-span [11]. Further research is needed to better understand the relationship between SWS and pituitary macroadenomas. Studies involving larger patient cohorts with SWS are necessary to establish the true prevalence of this association and identify any common genetic or molecular factors. Additionally, investigating the hormonal and developmental aspects of this potential link may provide valuable insights into the underlying mechanisms.

## Conclusion

While the association between SWS and pituitary macroadenomas is not well-established, it warrants attention in the clinical setting. Although the current evidence is limited, the possibility of a true association should not be dismissed. Given the rarity of both conditions, their coexistence could suggest a shared pathophysiological mechanism rather than mere coincidence. However, due to the limited number of reported cases, it is also plausible that the observed association is coincidental. Healthcare providers caring for individuals with SWS should be aware of the potential risk and consider appropriate evaluations, including neuroimaging and endocrine assessments, to detect and manage pituitary tumors in a timely manner. Further research is needed to clarify the underlying mechanisms and provide evidence-based guidelines for screening and management in this unique patient population. Large-scale studies and detailed case reviews will be essential to determine the true nature of this association and to develop robust clinical protocols.

## Ethics approval

Not applicable.

## Author contributions

**Razi Hashmi:** Conceptualization, Writing - original draft, writing - review and editing. **Mohamed Elmeligy:** Overview of the manuscript. Writing - original draft, writing - review and editing. **Daniel Fabian:** Writing - original draft, writing - review and editing. **Arun Mahtani:** Writing - original draft, writing - review and editing. **Paul Karroum:** Writing - original draft, writing - review and editing. **Meena Farid:** Writing - original draft, writing - review and editing. **Gianpaolo Piccione:** Writing - original draft, writing - review and editing. **Meheret Kinfe:** Writing - original draft, writing - review and editing. **Mahmoud Mahmoud:** Providing Radiologic imaging with precise analysis/descriptions. **Mohamed Albakri:** Review and editing. **Inderbir Padda:** Final review, editing, and submission process.

## Patient consent

The authors of this manuscript have obtained written informed consent for publication of this article.
